# Intrathoracic splenosis presenting as persistent chest pain

**DOI:** 10.1186/1749-8090-7-84

**Published:** 2012-09-07

**Authors:** Shinichi Fukuhara, Samuel Tyagi, Jaime Yun, Martin Karpeh, Angelo Reyes

**Affiliations:** 1 Department of Surgery, Beth Israel Medical Center, Albert Einstein College of Medicine, 317 E.17th St., New York, NY, 10003, USA

**Keywords:** Intrathoracic splenosis, Splenic injury, Splenectomy, Diaphragmatic injury

## Abstract

Thoracic splenosis is a rare entity resulting from splenic and diaphragmatic injury. Patients remain asymptomatic, and surgical intervention is not indicated in the majority of cases. We report a case of a 50-year-old male with a history of splenectomy due to a gunshot wound 30 years previously who presented with vague, progressively worsening chest pain. He was found to have a large intrathoracic splenosis. Unique features of our patient include the presence of symptoms, the significant interval growth of the splenic tissue, and the unprecedented size of the mass, which we believe to be the largest among those previously described.

## Background

Thoracic splenosis is a rare, benign condition involving autotransplantation of splenic tissue into the pleural cavity via trauma or surgery. It is usually incidentally detected, asymptomatic, and therapy is not indicated. Due to the resemblance to malignancy on diagnostic imaging studies, the majority of patients have undergone extensive workups or invasive procedures in the past [1]. It has been suggested that removal of thoracic splenic tissue in patients without functional abdominal splenic tissue may render the patient asplenic, increasing the risk of infection [2], although this concept is still controversial. We herein describe a case of symptomatic intrathoracic splenosis in a 50-year-old man. To our knowledge, thoracic splenosis has not been associated with symptoms with the exception of three previously reported cases that involved pleuritic chest pain and recurrent hemoptysis [3-5].

## Case presentation

**Figure 1:**
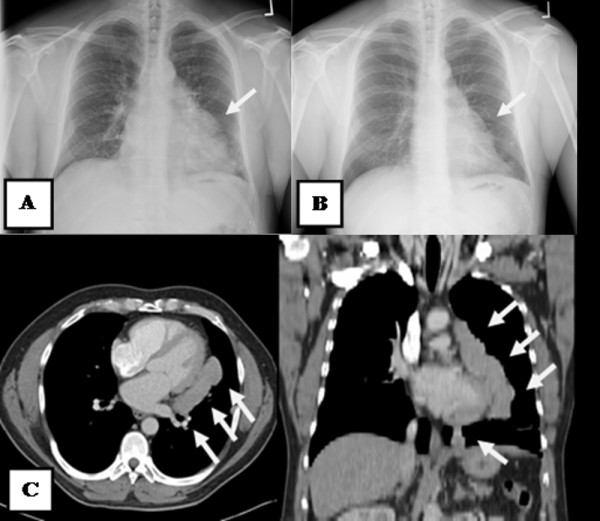
Chest X-rays (A) at presentation; (B) 1 year previously; (C) Computed tomography demonstrated a homogenously enhanced mass (arrows).

**Figure 2:**
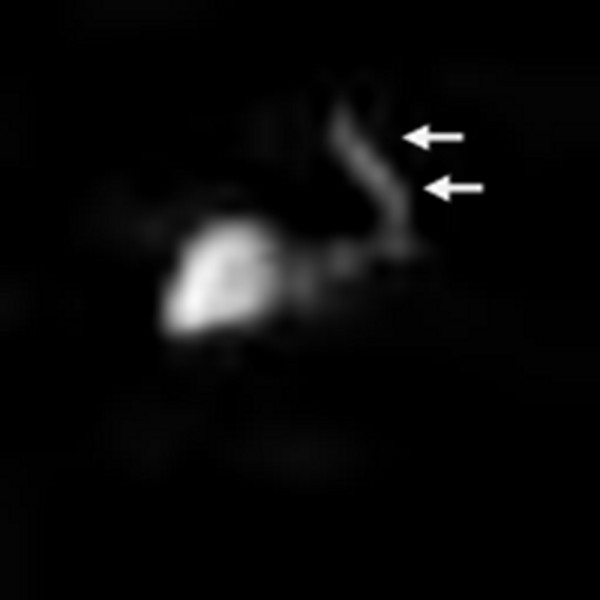
^**99m**^**Tc sulfur colloid scintigraphy (anterior view) showed liver uptake as well as the nodule uptake (arrows) corresponding to pleurocardial splenosis.**

**Figure 3:**
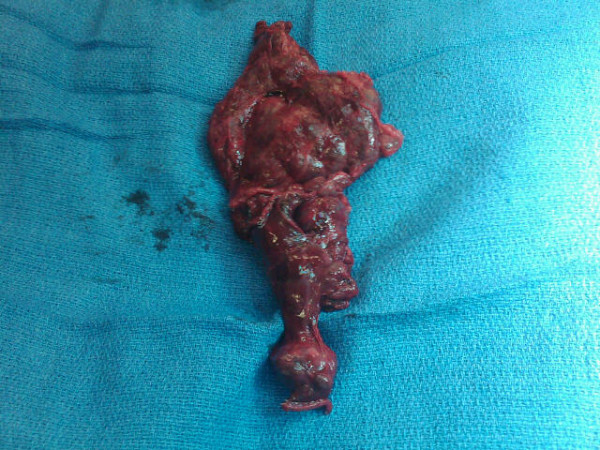
The specimen comprised multiple irregular, tan-pink, soft, multilobar tissues, including the following sizes, 11.5- × 9.0- × 1.5-cm (80 g, shown), 3.5- × 2.0- × 0.9-cm, 2.0- × 1.5- × 1.0-cm, and other multiple small fragments.

A 50-year-old male presented with a history of left-sided chest pain for a few years that had been progressively worsening over the last 3 months. The pain was characterized as vague and intermittent, although there were some fluctuations in intensity. He had a gunshot wound to the left upper abdomen resulting in diaphragmatic and splenic rupture 30 years ago. He stated that his spleen had been removed at that time. Other medical history included chronic hepatitis C and a 30 pack-year history of smoking. His physical examination revealed several abdominal scars, but was otherwise unremarkable. Routine blood work demonstrated no significant laboratory abnormalities. A peripheral smear was not obtained. On presentation, his chest X-ray showed a large, ill-defined, clearly marginated mass along the left heart border on the posteroanterior view. In comparison, the chest radiograph obtained 1 year previously was reviewed, and the lesion was noted, in retrospect, to have been present at that time; it had obviously grown over the 12-month period between the two examinations (Figures 1A and 1B). Computed tomography (CT) of the chest with intravenous contrast showed a large, homogeneously enhanced mass of 13 × 3 × 8 cm extending from the level of the aortic arch inferiorly along the mediastinal contour into the left upper quadrant, corresponding to the lesion seen on the chest radiograph (Figure 1C). Technecium-99 m (^99m^Tc) sulfur colloid scintigraphy was performed based on a high index of suspicion for splenosis given the patient’s trauma history and subsequent splenectomy. Thick linear uptake was present in the medial left hemithorax, corresponding to the mass on the chest CT, and was consistent with splenic tissue (Figure 2). Although the diagnosis of intrathoracic splenosis was established, the patient was referred to the thoracic surgery service because of symptoms refractory to pain control with analgesia as well as the interval growth of the mass. Video-assisted thoracoscopic surgery (VATS) was attempted. However, it was converted to an open thoracotomy due to excessive adhesions. A large anterior mediastinal mass extended up into the left hilum, overlying the entire aspect of the pericardium. In addition, there were multiple nodules along the surface of the lung and the diaphragmatic and left pleural surfaces. Notably, the phrenic nerve was completely surrounded by the mass. The phrenic nerve was dissected free of the mass and preserved. The mass and all remaining implants were fully mobilized and resected (Figure 3). The patient was discharged after an uneventful postoperative course. The chronic pain clearly improved at 6-month postoperative follow-up. The pathological report revealed splenic tissue.

## Discussion and conclusion

Thoracic splenosis, first reported by Shaw and Shafi in 1937 [6], describes the autotransplantation of splenic tissue into the pleural cavity after splenectomy for traumatic or iatrogenic injury, resulting in multiple nodular implants on the left pleura. Autotransplanted spleens differ from accessory spleens by blood supply, local perforator arteries versus splenic artery respectively [7]. Thoracic splenosis occurs less frequently than does abdominal splenosis and may be found in 18% of patients after splenic rupture. However, its frequency is likely underestimated because most splenic implants are asymptomatic and are only incidentally discovered during chest X-ray or CT. The average interval between initial trauma and diagnosis of thoracic splenosis is 18.8 years [5]. The splenic implants are sessile or pedunculated reddish blue nodules ranging from a few millimeters to 7 cm in diameter [8]. To our knowledge, the size of the present case is the largest among those in previously reported intrathoracic splenosis. The current diagnostic modality of choice for splenosis is noninvasive nuclear scintigraphy. ^99m^Tc sulfur colloid scintigraphy was first used to diagnose splenosis based on the ability of the radiolabeled colloid to localize in the reticuloendothelial system. However, scintigraphy using ^99m^Tc heat-damaged erythrocytes or indium 111-labeled platelets is more sensitive and specific for splenic uptake, making these tests the current diagnostic tools of choice [9]. It is suggested that implanted splenic tissue offers some degree of protection against bacterial infection, lowering the frequency of postsplenectomy sepsis [1], although the degree of immunoprotection offered by this tissue remains unclear. Many of the human data are in the form of case reports documenting failure of splenic tissue to protect against septicemia [9], possibly because of the small amount of tissue as well as poor vascularization. A review of the English literature revealed only three symptomatic patients with intrathoracic splenosis: two who presented with hemoptysis and one who presented with pleuritic chest pain [3-5]. We postulate that the pain in our patient was referred pain due to a mass effect and irritation of the pericardium, parietal pleura, and diaphragm mediated by the phrenic nerve. In addition, the mass was obviously growing as shown in the chest X-ray, and this mass effect can explain the recent exacerbation of the chest pain. The natural history of intrathoracic splenosis is essentially benign. However in our case, the autotransplanted tissue increased in size significantly. The diagnosis of thoracic splenosis can be established noninvasively with several diagnostic modalities, and surgical intervention is not indicated unless the patient is symptomatic. In cases of symptomatic thoracic splenosis refractory to non-operative treatment, surgery is beneficial.

## Consent

Written informed consent was obtained from the patient for publication of this case report. A copy of written consent is available for review by the Editors-in Chief of this journal.

## Competing interests

The authors declare that they have no competing interests.

## Authors’ contribution

SF and ST wrote the manuscript. AR, JY and SF performed surgery. MK supervised manuscript and entire treatment. All Authors read and approved the final manuscript.
